# Editorial: Dynamic functioning of resting state networks in physiological and pathological conditions, volume II

**DOI:** 10.3389/fnins.2022.1134113

**Published:** 2023-01-18

**Authors:** Roberto Esposito, Nicoletta Cera, Fernando Barbosa, Filippo Cieri

**Affiliations:** ^1^Titano Diagnostic Clinic, Falciano, San Marino; ^2^Azienda Sanitaria Territoriale (AST), Pesaro-Urbino (PU), Marche, Italy; ^3^Faculty of Psychology and Education Sciences, University of Porto, Porto, Portugal; ^4^Coimbra Institute for Biomedical Imaging and Translational Research (CIBIT), Coimbra, Portugal; ^5^Department of Neurology, Cleveland Clinic Lou Ruvo Center for Brain Health, Las Vegas, NV, United States

**Keywords:** resting state networks, dynamic brain activity, default network, advanced neuroimaging techniques, neuropsychiatric diseases

## 1. Introduction

Advanced neuroimaging techniques represent a valid tool to study brain physiology and neural mechanisms in several pathological conditions such as neuropsychiatric disorders (Spinosa et al., 2022), allowing to examine brain structural and functional changes (Cieri and Esposito, 2018; Esposito et al., 2018; Cieri et al., 2020). This Research Topic is a second volume of a previous Research Topic, now convening nine research articles based on the current understanding of brain neuroimaging technique addressing theoretical and methodological questions.

## 2. Neurodegenerative diseases: Mild cognitive impairment and Alzheimer's disease

Wang et al. explored the clinical role of structural and functional MRI in early diagnose of Alzheimer's Disease (AD) and amnestic MCI (aMCI), where fMRI can identify brain functional abnormalities in the early stages of the disease. Combining the textural features of the amplitude of low frequency fluctuation (ALFF) in the slow-frequency band and structural images in the hippocampus, the authors investigated diagnostic performance of their approach for AD and aMCI, using multimodal radiomics technique. Radiomics models based on structural images in the hippocampus had a better diagnostic performance for AD compared with the models using ALFF, while the latest model exhibited better discriminant performance for aMCI than the structural approach.

## 3. Emotional disturbances and psychiatric diseases

Allen et al. examined whether psychopathic traits are associated with aberrant inter-network connectivity, intra-network connectivity, and amplitude of fluctuations across limbic and surrounding paralimbic regions among incarcerated women. PCL-R Factor 1 scores (interpersonal/affective psychopathic traits) were associated with increased low-frequency fluctuations in executive control and attentional networks, decreased high-frequency fluctuations in executive control and visual networks, and decreased intra-network functional connectivity in default network (DN). PCL-R Factor 2 scores (lifestyle/antisocial psychopathic traits) were associated with decreased high-frequency fluctuations and DN, and both increased and decreased intra-network functional connectivity in visual networks.

Zhang et al. explored neuropathological mechanisms of postpartum depression (PPD) through voxel-based degree centrality (DC) analysis to explore intrinsic dysconnectivity pattern of whole-brain functional networks in this clinical condition. DC image, clinical symptom correlation, and seed-based functional connectivity (FC) analyses were performed to reveal the abnormalities of the whole-brain functional network in PPD. Compared with healthy controls (HCs), patients exhibited significantly increased DC in the right hippocampus and left inferior orbitofrontal gyrus. In the seed-based FC analyses, the PPD showed significantly decreased FC between the right hippocampus and right middle frontal gyrus, between the right hippocampus and left median cingulate and paracingulate gyri, and between the left inferior orbitofrontal gyrus and the left fusiform (FFG.L) compared with HCs. The authors provided evidence of aberrant voxel-based FC within brain regions in PDD, potentially helpful to better understand neural circuitry dysfunction in these patients.

## 4. Other clinical conditions

### 4.1. Epilepsy

Qin et al. explored idiopathic generalized epilepsy and particularly the dynamics and the causal relationship among 3–6 Hz generalized spike-wave discharges and extensive altered interactions in subcortical-cortical circuit, using rs-fMRI. Their results showed that thalamus and precuneus were key regions representing abnormal FC in subcortical-cortical circuit. Moreover, the connectivity between precuneus and adjacent regions had causal effect on the widespread dysfunction of the thalamocortical circuit, and the connection between the striatum and thalamus indicated the modulation role on the cortical connection in epilepsy.

Li et al. focused their attention on neural mechanisms underlying the alterations of thalamus in children with generalized tonic-clonic seizures. They explored the temporal properties of functional pathways connecting thalamus in these patients. The findings of both increased and decreased connectivity variability in the thalamo-cortical network imply a dynamic restructuring of the functional pathways connecting the thalamus in children with generalized tonic-clonic seizures. These results contribute to extend the understanding of the neural mechanism underlying this disorder in children.

### 4.2. Rheumatoid arthritis

Fanton et al. explored time-varying changes in brain network integration and segregation during pain over a disease-affected area (joint) compared to a neutral site (thumbnail) in patients with rheumatoid arthritis (RA). The authors quantified measures of integration and segregation at multiple spatial scales, both at the level of single nodes and communities (clusters of nodes), finding that Participation Coefficient (PC) at the community level was generally higher in patients compared to HCs during and after painful pressure over the inflamed joint and corresponding site in controls. This shows that all brain communities integrate more in patients than in HCs for time points following painful stimulation to a disease-relevant body site. Moreover, there was no specific nodal contribution to brain network integration or segregation. Altogether, this evidence suggests widespread and persistent changes in network interaction in RA patients compared to HCs in response to painful stimulation.

### 4.3. Kallmann syndrome

Di Nardo et al. investigated the dynamic spectral changes of the sensorimotor network FC in Kallmann syndrome (KS) patients with and without mirror movement (MM) symptom. Compared to KS patients without MM and HCs, the sensorimotor brain network (SMN) of patients with MM displayed significantly larger spectral power changes in the slow 3 canonical sub-band and significantly fewer transitions between state 1 (less recurrent) and state 2 (more recurrent). This study shows that the presence of mirror movement in this syndrome is associated with reduced spontaneous transitions of the SMN between dynamic FC states and a higher recurrence and an increased spectral power change of the high-frequency state.

### 4.4. Pathological fatigue

Skau et al. recruited individuals suffering from pathological fatigue after mild traumatic brain injury (mTBI). They used functional near-infrared spectroscopy to assess hemodynamic changes in the frontal cortex. The participants underwent to a session before and after an experiment involving cognitive tasks, including the Digit Symbol Coding test. The authors have shown a Group vs. Time interaction with a *post-hoc* test revealing that patients developed higher modularity toward the end of the cognitive test session. This work helps to identify how functional networks differ under pathological fatigue compared to HCs.

## 5. Brain physiology and methodological approaches

Vazquez-Trejo et al. have used connectotyping, which efficiently models functional brain connectivity to reveal the progression of temporal brain connectivity patterns in task fMRI. They found significantly different dynamic connectivity patterns during word vs. pseudoword processing between the Fronto-Parietal and Cingulo-Parietal Systems, that are involved in cognitive task control, memory retrieval, and semantic processing. The findings support the presence of dynamic changes in functional connectivity during task execution and that such changes can be characterized using connectotyping.

## Author contributions

RE, FC, NC, and FB conceived and developed the Research Topic. RE and FC wrote the editorial. All authors contributed to the article and approved the submitted version.
